# Genetic diversity and biogeography of *T*. *officinale* inferred from multi locus sequence typing approach

**DOI:** 10.1371/journal.pone.0203275

**Published:** 2018-09-18

**Authors:** Mohammadjavad Jafari, Waheed Akram, Yanju Pang, Aqeel Ahmad, Shakeel Ahmed, Nasim Ahmad Yasin, Tehmina Anjum, Basharat Ali, Xiangdong Hu, Xiaohua Li, Shuang Dong, Qian Cai, Matteo Ciprian, Monika Bielec, Sheng Hu, Fatemeh Sefidkon, Xuebo Hu

**Affiliations:** 1 Laboratory of Drug Discovery and Molecular Engineering, Department of Medicinal Plants, College of Plant Science and Technology, Huazhong Agricultural University, Wuhan, China; 2 National-Regional Joint Engineering Research Center in Hubei for Medicinal Plant Breeding and Cultivation, Huazhong Agricultural University, Wuhan, China; 3 Medicinal Plant Engineering Research Center of Hubei Province, Huazhong Agricultural University, Wuhan, China; 4 Institute of Agricultural Sciences, University of the Punjab, Lahore, Pakistan; 5 Department of Microbiology and Molecular Genetics, University of the Punjab, Lahore, Pakistan; 6 Hubei Cancer Hospital, Wuhan, China; 7 Laboratory of Organometallics, Catalysis and Ordered Materials, State Key Laboratory of Advanced Technology for Materials Synthesis and Processing, Wuhan University of Technology, Wuhan, China; 8 Department of Biomedical Engineering, Wuhan University of Technology, Wuhan, China; 9 Research Institute of Forest and Rangeland, Iran; National Cheng Kung University, TAIWAN

## Abstract

*Taraxacum officinale* (Asteraceae) is widely distributed weedy plant used as a traditional medicinal herb. The population genetics and historical biogeography of this plant have remained relatively unexplored. This study explores phylogeny, population genetics and ancestral reconstructions adopting multi locus sequence typing (MLST) approach. MLST sequences dataset was generated from genomics and chloroplast DNA sequences obtained from 31 *T*. *officinale* haplotypes located in 16 different countries. Phylogenetic analysis distributed these haplotypes in well differentiated geographic clades. The study suggested a close relationship between Europe and adjacent Asian countries. Populations of these regions predominantly formed common haplogroups, showed considerable level of gene flow and evidence for recombination events across European and Asian population. Biogeographical inferences obtained by applying statistical dispersal-vicariance analysis (S-DIVA) and Bayesian binary MCMC (BBM) analysis showed that *T*. *officinale* was putatively originated in Europe. Molecular clock analysis based on ITS dataset suggested that the divergence between Europe and East Asian populations can be dated to 1.07 Mya with subsequent dispersal and vicariance events. Among different spatial process long distance seed dispersal mediated by wind had potentially assisted the population expansion of *T*. *officinale*.

## Introduction

Dandelion (*Taraxacum officinale*; *Compositae-Lactuceae*) is a group of highly invasive wild plants with worldwide distribution. *Taraxacum* species dominate in towns, along roadsides, shores of conduits and in many other rural sites. The representative species of this genus are *Taraxacum officinale* and *T*. *erythrospermum*. *T*. *officinale* has been documented particularly in North America, Asia, and Europe [1]. In USA, *T*. *officinale* is found abundantly in cold regions. This indicates that it is basically acquainted with America through Alaska [2]. It was reported that the spread of *T*. *officinale* was contributed by the early European pilgrims who introduced different species of dandelion as a fancy [3, 4]. Ecological variation in *T*. *officinale* is derived from relatively minor morphological modifications, leaving a seemingly limited set of morphological characters for phylogenetic studies. Multiple hybridization events in the evolution process of *Taraxacum* impair great difficulties in the interpretation of specific variation in *Taraxacum* phylogeny and biogeography [5]. A remarkable phenomenon is common existence of geographical parthenogenesis [6]. Another situation worth mentioning is the occurrence of several geographically restricted complexes of sympatric diploid sexuality and closely related agamosperms comprising higher levels of variations and dynamics [7]. These processes are discussed in more detail by Kirschner et al., 2003 [8].

The molecular typing scheme should generate data that can be employed to resolve the structure of the population in addition to provide highly discriminating data and resolving biological relationships among collection of isolates. Molecular data from a single barcode locus is not sufficient for a broader context exploration of phylogeny. A larger number of loci would be preferable to explore population genetics [9]. Multi locus sequence typing (MLST) implies a typing scheme that involves the determination of the alleles at multiple loci by nucleotide sequencing. This method have been employed in studies of the population biology, pathogenicity, and evolution [9]. Phylogenetic analysis based on combined dataset (MLST) from nuclear (nrDNA) and chloroplast DNA (cpDNA) loci has been successfully used for some organisms [10–12].

Utilization of sequence variations of ITS region of genomic DNA (nrDNA) alone is not suitable for phylogenetic analysis of *T*. *officinale* because of high sequence variations with in agamospermous polyploid individuals [13]. Congruence of homologous characters emerges from the interaction of coding and non-coding molecular barcode datasets that allows overall phylogenetic resolution [14]. Therefore, this combined dataset analysis approach (MLST) can uncover underlying homology and detailed phylogeny to understand *Taraxacum* evolution.

Spatial processes are crucial for determining the structure and dynamics of population and communities [15]. Seed dispersal is the premier spatial demographic process among sessile organisms. The seed-dispersion pattern not only determines the potential area of plant recruitment but also affects the rates of gene flow thus, influences genetic structure of population [16]. Wind mediated long-distance dispersal events play a major role in determining population spread, the flow of individuals between population and the colonization of unoccupied habitats [17, 18]. *Taraxacum* is the most popular example for a wind-dispersed species with a hairy structure aiding aerial transport. The vast majority (99%) of wind-dispersed seeds are predicted to travel up to two meters [19]. Together with the molecular data, the knowledge of spatial processes promises more mechanistic understanding of population dynamics and biogeography.

In this study, samples of *T*. *officinae* were collected from 16 different countries of the world and molecular data from nrDNA and cpDNA barcode loci combined. We constructed topologies based upon combined datasets of barcode loci and performed haplotype and recombination analysis to uncover the phylogeny, evolution process and current geographical distribution patterns in the level of species.

Furthermore, a historical biogeography model of *T*. *officinale* was constructed by applying statistical dispersal-vicariance analysis (S-DIVA) and Bayesian binary MCMC (BBM) analysis implemented in RASP (Reconstruct Ancestral State in Phylogenies) and linking outcomes of this model with spatial process. It is proposed that wind-mediated seed dispersal could have played an important role in the diversification of *T*. *officinale*. Congruence of *T*. *officinale* population history with some climatic events illustrate the relevance of spatial process for determining the dynamics of population and historical biogeography of *T*. *officinale*. Previous studies have mostly focused on phylogeny of *T*. *officinale* [8, 13, 20]. To best of our knowledge, no any specialized study has described evolution and biogeography of *T*. *officinale* by using MLST approach and spatial processes.

## Materials and methods

### Dandelion sample collection

Leaf samples of *T*. *officinale* were collected from various locations of China or donated by researchers in the respective countries. Taraxacum plants are highly invasive and can be found abundantly on roads sides, gardens and rural areas. No special permissions were required for collection of plant samples. All samples were totally dried to null the propagation capability before shipment. Therefore, no special permissions were required for shipment and transportation process. This study did not involve endangered or protected species. Foreign samples were donated by samplers themselves or by the researchers from Department of Agriculture, Azad University of Gorgan, Iran; Institute of Agricultural Sciences, University of the Punjab, Pakistan. Details of sampling localities are shown in supplemental table A in S1 Tables.

### DNA isolation

For fresh leaf samples, 50–100 mg of leaf material was crushed in liquid nitrogen. Genomic DNA was then extracted using the Sangon Biotech Plant DNA extraction kit (Shanghai, China) following manufacturer’s instructions. For dried leaf samples, leaf material was superficial cleaned through moderate friction using absorbent paper. DNA was extracted by adopting a modified CTAB method [21]. The quantity and quality of extracted DNA were determined by nano-drop spectrophotometer (NanoDrop 2000, Thermo Scientific).

### PCR amplification and DNA sequencing

A pilot study was performed to select DNA barcode loci showing more than 90% PCR amplification efficacy. Afterwards, 4 candidate DNA barcode loci were amplified and sequenced from the extracted DNA of the leaf samples. These included two coding cpDNA loci ‘matK and rbcL’, one non-coding cpDNA intergenic spacer loci ‘psbA-trnH’ and a nuclear DNA locus ‘ITS2’ (Table 1). Previously established primers were used to make PCR amplifications of these selected barcode loci. Details of the primers were listed in supplementary table B in S1 Tables. PCR was performed in a reaction volume of 30 μL, using Sangon Biotech (Shanghai China) nTaq 2X reaction mixture. PCR amplifications were obtained on A100 ThermoCycler (LongGene, China) using 20 ng of template genomic DNA. Amplified products were inspected by electrophoresis on 1% agarose gel prior to DNA sequencing.

**Table 1:** The profile of DNA barcode loci for the selected *Taraxacum* samples.

Parameter assessed	DNA barcode locus			
	matK	rbcL	ITS2	psbA-trnH
Number of samples	70	70	70	70
PCR Success rate	100%	97%	100%	93%
Sequencing Success rate	100%	100%	100%	100%
Sequence Length (bp)	699–761	529–637	427–472	403–431
Aligned Length (bp)	761	637	472	431
Numbers of Variable sites	86	78	131	56
Parsimony informative sites	43	37	56	29

### Sequence variation and population genetic structure

DNA sequences were aligned using Clustal X2 [22] and converted in FASTA format for further analysis. The data was used individually and also in combination of barcode loci (MLST approach) to generate genetic distance, species discrimination and phylogenetic tree construction. Inter- and intra-specific genetic distances were calculated according to Kimura 2-parameter model [23] in MEGA 7 software [24]. The accuracy of species discrimination for each DNA barcodes was calculated using “best match”, “best close match”, and “all species barcodes” criteria as proposed previously [25]. Genetic diversity, recombination analysis and neutrality tests were performed in DNAspV5 [26].

Haplotype networks were generated to depict relationship among *Taraxacum* population belonging to different geographical regions with PopArt (http://popart.otago.ac.nz). Networks were constructed under 95% parsimonious branch connections. Fu’s F*s* and Tajima’s *D* approaches were used to collect information regarding demographic history of *Taraxacum* population [27, 28].

Potential recombinants along with parental analysis and approximation of possible recombination breakpoint positions in *T*. *officinale* haplotypes were analyzed by RDP version 4 [29]. Recombination detection was performed using GENECONV [30], Maximum Chi Square [31] Chimaera [32, 33] and SisterScan [34] methods with default settings. Any potential recombinant event detected by two or more methods were used in this study. A breakpoint map containing the positions of all clearly identifiable breakpoints was compiled after discovery of a set of unique recombination events.

To support our biogeographical model, we estimated pairwise population differentiation using Slatkin’s linearized FST [35] and the absolute number of migrants (Gene flow) between population using Arlequin ver. 3.0 software [36]. The results were generated from 10,000 random permutations as described [37].

### Phylogenetic analyses and divergence time estimation

Phylogenetic inferences between haplotypes were generated using Maximum Likehood (ML) method in MEGA 7 [24]. Every haplotype in the tree was represented by all barcode loci. The best-fitted model of sequence evolution was tested in TOPALi (http://www.topali.org/) and the model parameter values were used to construct ML tree. Clade support was estimated using 1000 bootstrap pseudo replicates [38].

Divergence time was estimated using a Bayesian approach with BEAST software version 1.6.1 [70] using ITS datasets. Two runs of 108 chains were conducted, sampling every 1,000 generations. The settings used were the Yule tree prior, the HKY substitution model, three gamma categories and strict molecular clock. ITS substitution rate for asteraceae is 2.51x10-9 substitutions per site yer^-1^ based on *Eupatorium* spp. [23]. The best-fit model of sequence evolution was chosen by TOPALi as mentioned earlier. Tracer 1.5 (http://beast.bio.ed.ac.uk/Tracer) was used to check for convergence of the Markov chains and adequate effective sample size. A maximum clade credibility tree was obtained using Tree-Annotator software.

### Biogeographic analyses

The statistical dispersal-vicariance analysis (S-DIVA) and Bayesian binary MCMC (BBM) were implemented to infer ancestral distribution using RASP 3.2 (Yu et al. 2015). The trees previously constructed by BEAST analysis were used as the input data. Four regions were defined according to the sampling and distribution range of Taraxacum; Eur = Europe, WA = West Asia (Iran and Iraq), SA = South Asia (Pakistan), EA = East Asia (China, Korea). The MCMC chains were run for 5 × 10^6^ generations in all analyses. The data were sampled every 1000 generations, and the temperature for heating the chains was 0.1.

## Results

### PCR amplification and DNA sequencing of *Taraxacum* samples

*T*. *officinale* samples were collected globally from 33 different sites across two continents. Four DNA barcoding loci were selected after performing pilot studies. These loci were successfully amplified for all plant samples. In accordance with the previous knowledge, all four loci used in this study are considered highly polymorphic in nature [12]. Here 132 barcode sequences were produced. “Genome Workbench” software was used to perform BLAST analysis for molecular identification of plant samples based on these amplified loci. An alignment was generated using CLUSTAL W, covering four barcode genes for different *T*. *officinale* samples. This alignment was used for detection of recombination, construction of phylogenetic trees and haplotype networks. All the loci were amplified three times to get the best close match. Details of PCR amplifications are provided in Table 1.

### Interspecific genetic variations

After sequencing of all four DNA barcode loci, the aligned sequence lengths ranged from 427 to 452 bps. The number of variable sites ranged from 16 to 37 bases in the aligned gene sequences. On average basis, 6.3% nucleotide positions varied across all samples of *T*. *officinale*. The intra-specific distance ranged between 0.08 to 0.94% with an average value of 0.46% (Supplementary Table C in S1 Tables). Both gaps and insertions were seen in sequences of these barcode loci when aligned together for further analysis. Generally, ITS2 and psbA-trnH exhibited highest mean intra-specific variations respectively (Supplementary Table C in S1 Tables).

### Species identification and discrimination

**Fig 1:**
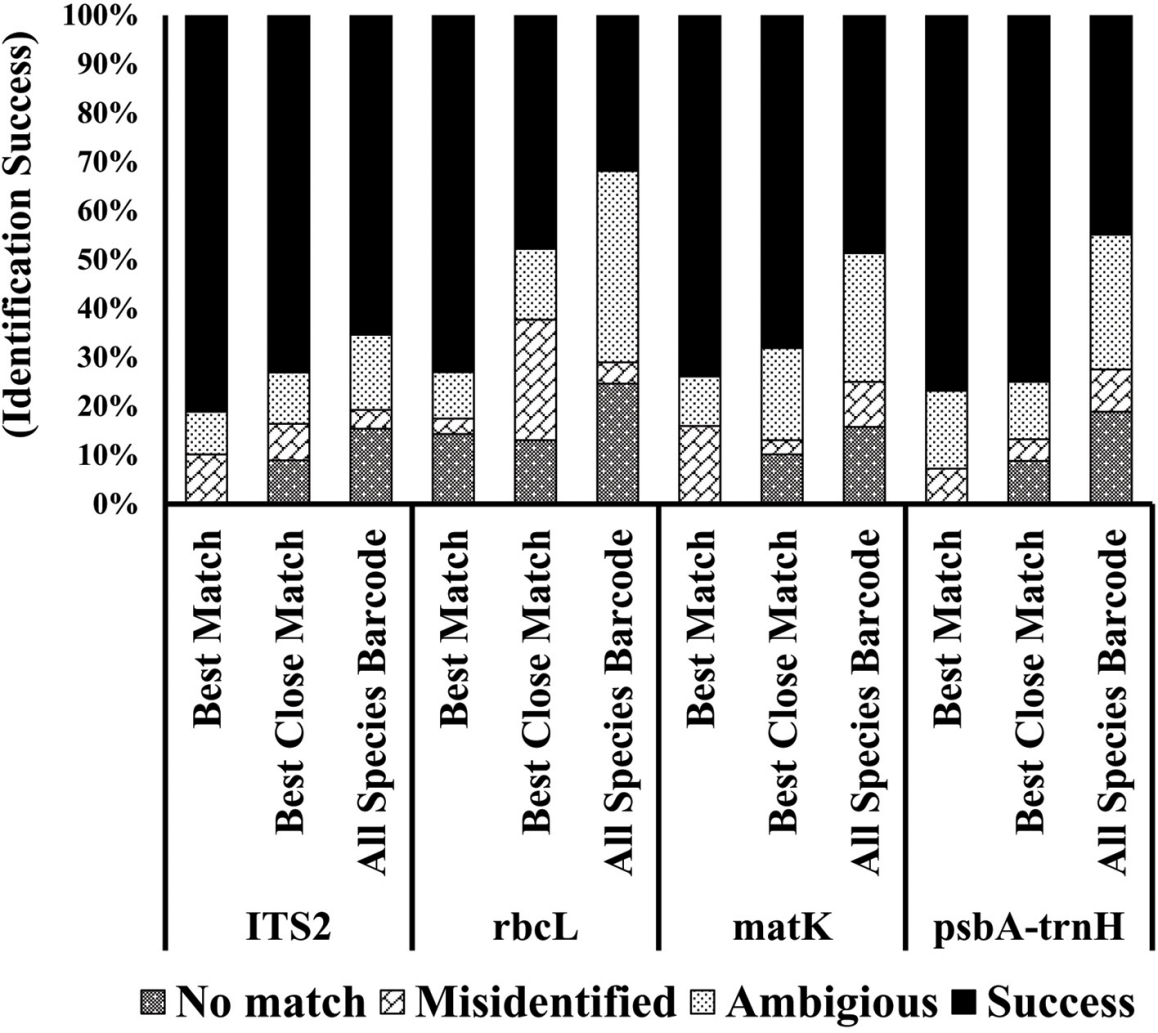
The sequence identification success rates of different barcode loci. Criteria for best match, best close match and all species barcode is based on Meier et al., 2006.

Parameters developed by [25] were used to search the best match and the best close match. These loci (ITS2, matK, rbcL and psbA-trnH) showed the best close match of 67.3, 58.6, 47.1 and 63.5% respectively (Fig 1). While performing the barcode analysis, these individual barcode candidate loci showed relatively less success rate as compared to the best close match and the best match (Fig 1). ITS2 had the highest success rates in the barcode analysis (Fig 1).

### Phylogenetic analysis

**Fig 2:**
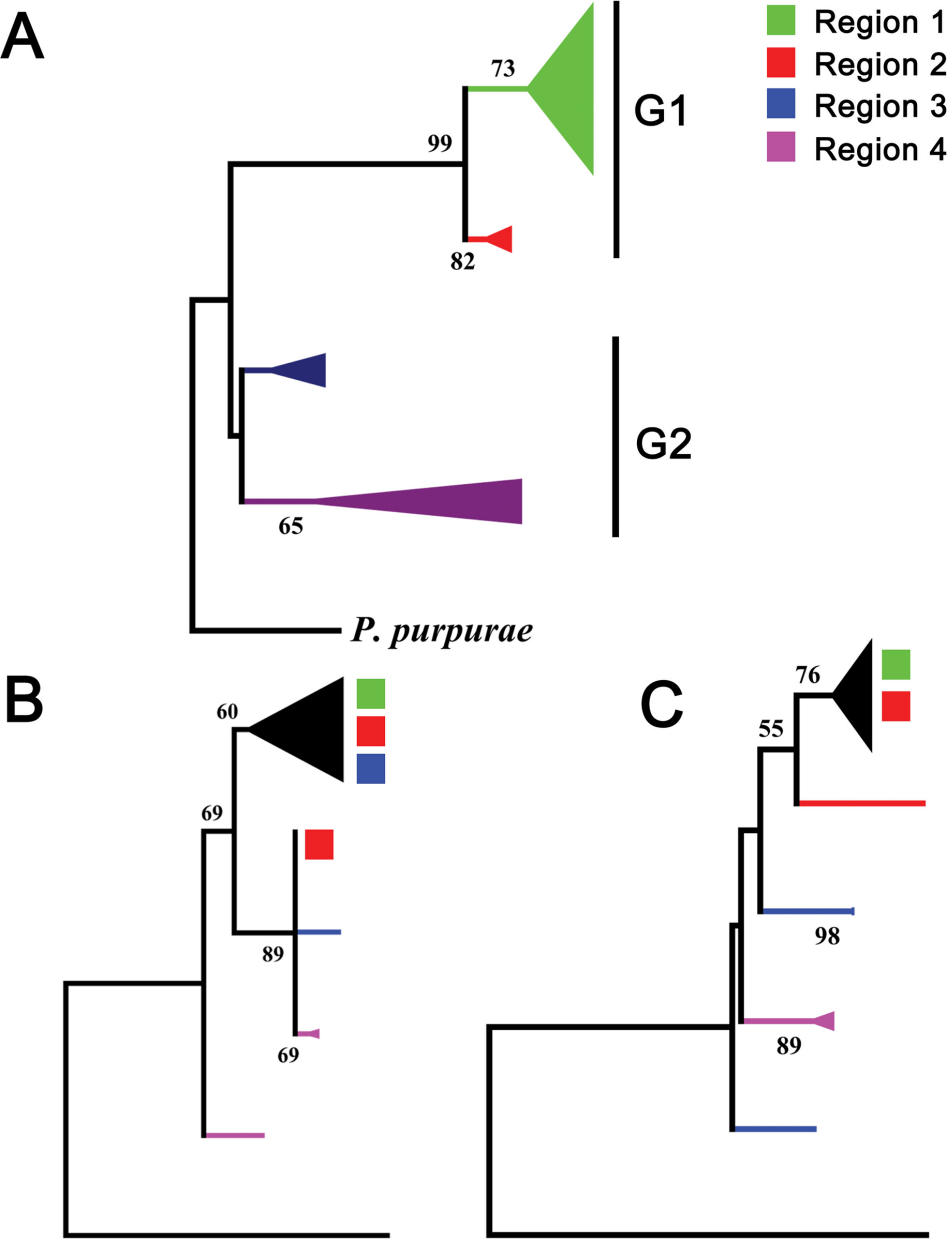
A maximum likehood phylogenetic tree estimated from four different barcode loci. Phylogenetic trees were inferred from either dataset of all barcode loci (A) of by using ITS (B) and cp DNA (C) datasets alone. Values on nodes indicate boot strap support. Region 1 = East Asia (China and Korea). Region 2 = South Asia (Pakistan), Region 3 = West Asia (Iran and Iraq), Region 4 = Europe. The size of triangle is proportional to the number of haplotypes.

We used the maximum likehood (ML) approach to obtain a consistent phylogeny based on nrDNA and cpDNA datasets. *P*. *purpurae* was used as out-group in topology construction. All the gene sequences were combined in the form of one dataset (multi locus sequence typing data ‘MLST’ approach) to provide an accurate phylogeny for the construction of phylogenetic trees. Haplotypes of different geographical origin fell into clearly separated clades (Fig 2). Tree branches mostly showed high bootstrap values. Some members with erratic behavior negatively affected the bootstrap value of occupied branches. Data obtained from individual DNA barcode genes was not sufficient to obtain deep phylogeny and varying specific details of relationship and related perspectives were seen in ML based phylogenetic trees based on individual barcode gene sequences (Fig 2).

All the clades on ML tree can be clearly divided into two main groups “1 and 2”. Group 1 contained haplotypes of East and South Asian countries (Fig 2). Group 2 was containing haplotypes from Europe and West Asian countries like Iran and Iraq.

### Population biology and phylogeographic structure

**Fig 3:**
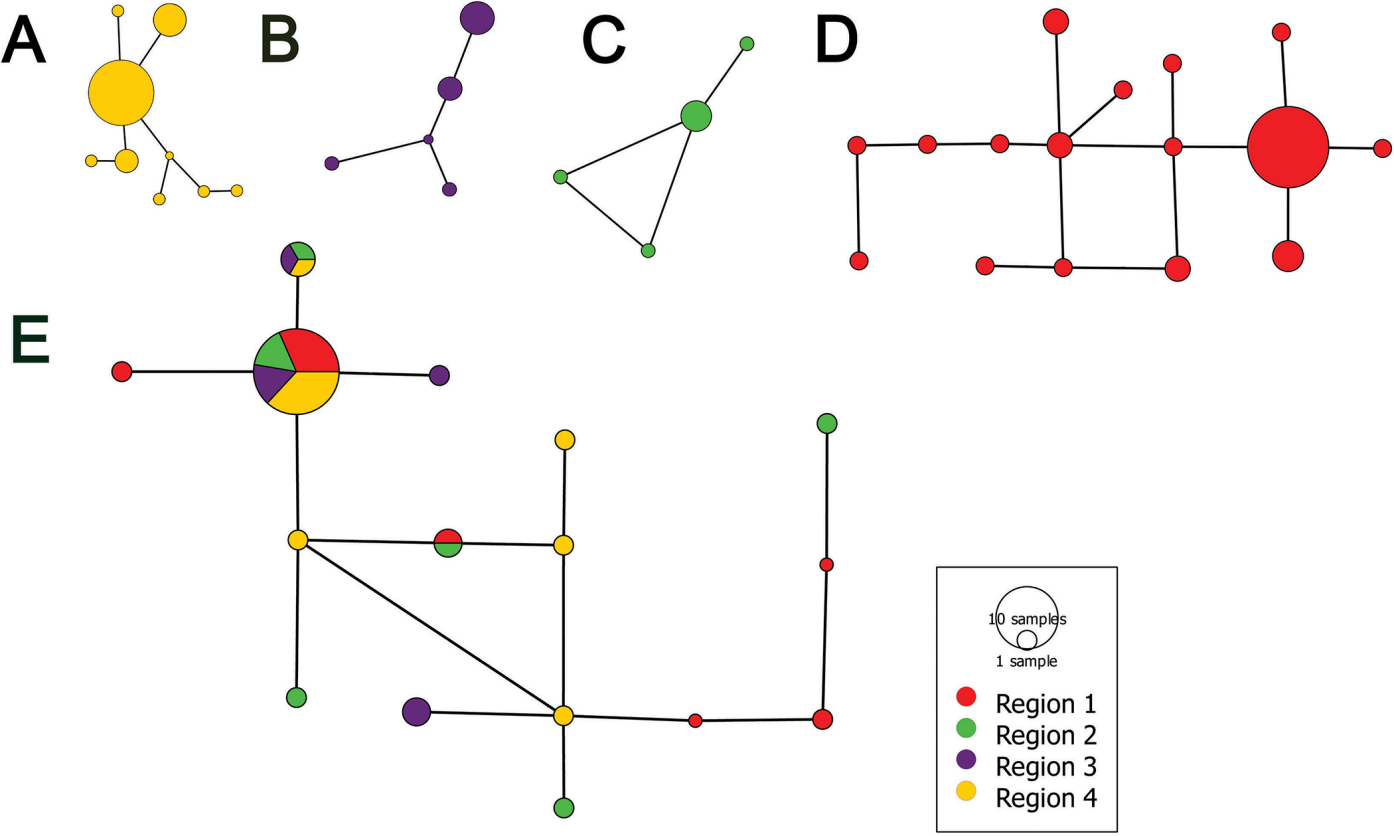
Haplotype analysis of populations of *T*. *officinales* belonging to different geographical origins. The analysis was performed using “PopART” software based on ‘Minimum Spanning Network’ approach. Haplogroups were made based on datasets of all four barcode loci. A = Haplotype network of samples belonging to region 4 (Europe). B = Haplotype network of samples belonging to region 3 (West Asia). C = Haplotype network of samples belonging to region 2 (South Asia). D = Haplotype network of samples belonging to region 1 (East Asia). E = Network of haplotypes belonging to all four sample regions.

From all *T*. *officinale* samples, 31 haplotypes were identified with some singletons and the remaining contained more than one (Fig 3). Haplotype diversities varied from 0.24–0.54 with nucleotide diversity ranging from 0.023 to 0.35 (Table 2). Furthermore, Tajima's D and Fu’s Fs tests were employed to detect deviation from neutrality (Table 2). These results demonstrate that population differentiation of *T*. *officinale* was driven by different selective forces and evolutionary processes. Positive values of these tests provide evidence of balancing selection or of a population subdivision; whereas, negative values indicate positive selection and demographic history of *T*. *officinale*.

**Table 2:** The nucleotide polymorphism of *Taraxacum* population.

Region	n	H	nd	hd	Tajima’s *D*	Fu’s F*s*
Europe	28	H1-H09	0.283	0.54	-1.79 (*P*<0.05)	-4.863 (*P*<0.01)
West Asia	13	H10-H14	0.078	0.31	-0.58 (*P*>0.10)	-1.07 (*P*<0.05)
South Asia	19	H15-H18	0.023	0.24	0.22 (*P*>0.10)	-0.08 (*P*>0.10)
East Asia	38	H18-H31	0.358	0.39	-0.82 (*P*>0.10)	-3.98 (*P*<0.01)

n = Number of samples, H = Haplotypes, hd = the haplotype diversity, nd = the nucleotide diversity, *p*-values are indicated in parenthesis

**Fig 4:**
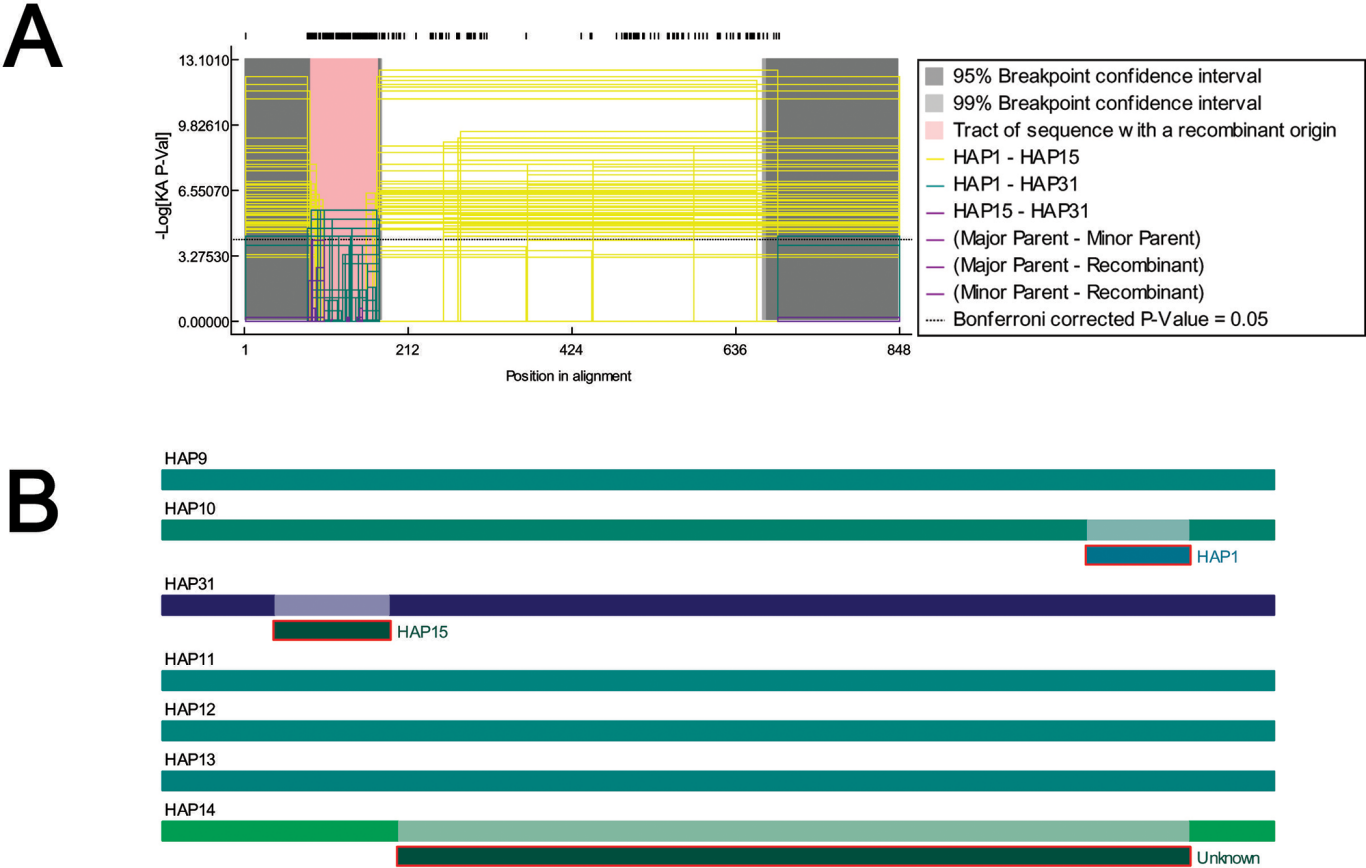
Recombination analysis among different population of *T*. *officinale*. Analysis was performed in software “RDP version 4”. A = GeneCov plot of potential recombination events along with putative parents. The black blocks above the plot represents positions of informative alleles. KA-P value represents P value based on Blast-Like Karlin-Altschul & Permutation analysis. Colored lines inside plot represents scorings of recombinants. B = An overview of recombination segments among different haplotypes of *T*. *officinale*.

Typing of *T*. *officinale* plant samples originated from several countries revealed the presence of some recombination events in members of different geographical origins (Fig 4). The permutation test indicated that the distribution of breakpoints was significantly (Fig 4). Different recombination events were detected by the selected recombination detection methods (Fig 4). The recombination analysis showed the exchange of genetic material between members of Europe and Asian lineages. Details of recombination events are provided in supplementary table E in S1 Tables.

The gene flow analysis was performed using sequences datasets of *Taraxacum* species. Relatively lower genetic differentiation (F_*ST*_) was seen between population of Europe and Asian regions with some non-significant indices (Table 3). Accordingly, considerable gene flow was seen between *Taraxacum* populations of Europe and Asian regions. The number of migrants (gene flow) among these populations ranged from 0.18 to 3.75 (Table 3).

**Table 3:** Pairwise genetic differentiation (FST) of *T*. *officinale* populations of different regions (below diagonal) and gene flow analysis among different populations (above diagonal).

Regions	Region 1	Region 2	Region 3	Region 4
Region 1	-	2.64	0.18	0.78
Region 2	0.04	-	1.62	3.75
Region 3	0.18	0.23*	-	1.28
Region 4	0.39*	0.08	0.28*	-

Region 1 = Europe, Region 2 = West Asia, Region 3 = South Asia, Region 4 = East Asia.

* = P<0.05

### Historical biogeography and divergence time estimates

**Fig 5:**
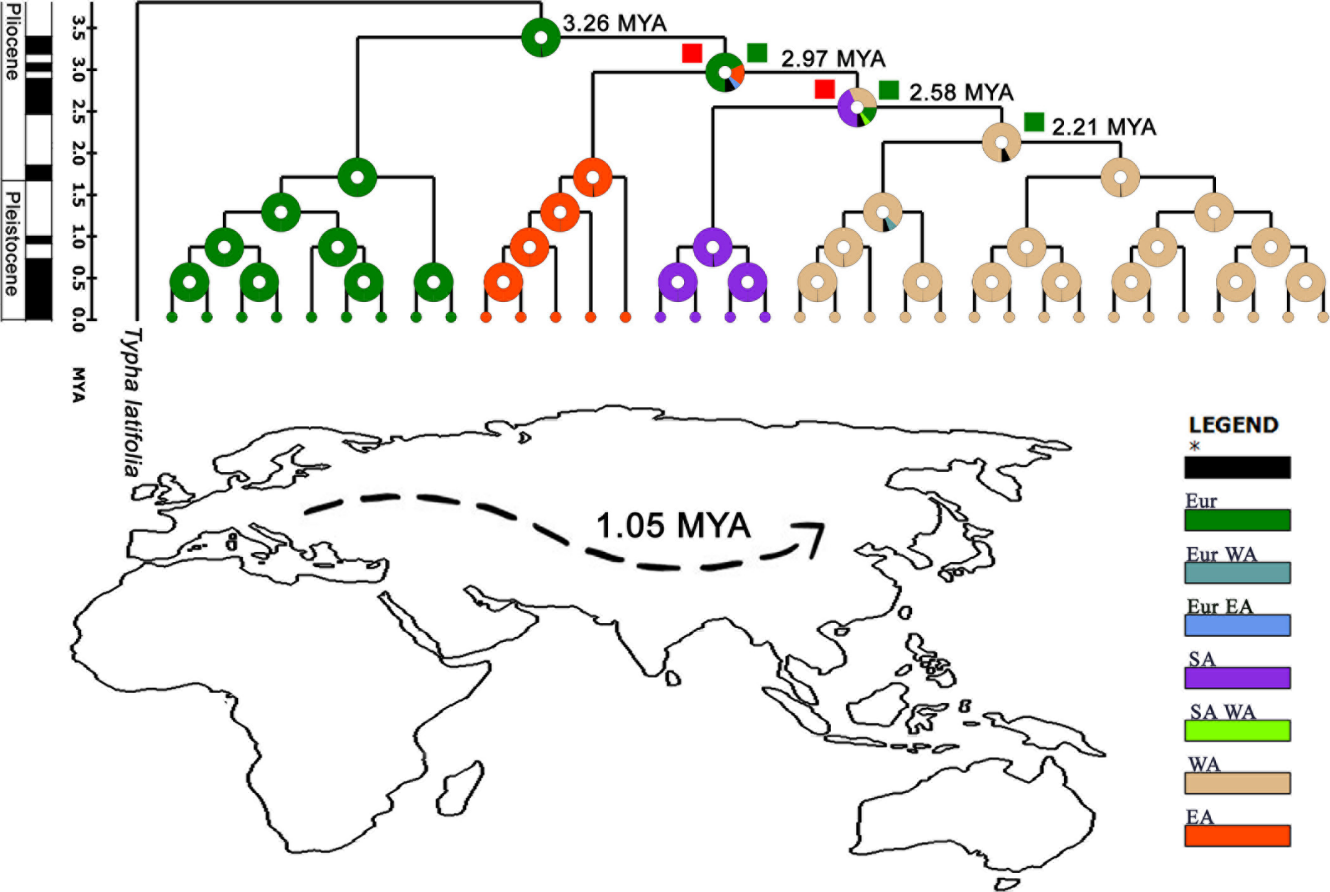
Historical biogeography of *T*. *officinale* and ancestral area reconstruction. A = Reconstruction of the ancestral area of *T*. *officinale* and related taxa. The pie charts at each node were obtained using S-DIVA and Bayes MCMC analysis. Upper case letters represent different regions: Eur = Europe. SA = South Asia (Pakistan), WA = West Asia (Iran, Iraq) EA = East Asia (China, Korea), Green square boxes represent dispersal events and red represents vicariance. B = Putative dispersal route of *T*. *officinale* as inferred from S-DIVA and Bayes MCMC analysis.

Lastly, biogeography of *T*. *officinale* was constructed by performing S-DIVA and Bayesian binary MCMC analyses (Fig 5). The root node of *T*. *officinale* is situated in Europe with subsequent dispersal and vicariance events between the ancestral and areas of present distribution. Some previous records also confirm that *T*. *officinale* is originated from Europe [39]. RASP inferred biogeography of *T*. *officinale* was also supported by rest of the analysis (phylogeny, haplotype, recombination events, parentage and gene flow analysis). Some potential haplotypes were detected with major and minor parents from Europe and Asia. Furthermore, higher gene flow was observed among population of Europe and Asia. These facts support as Europe is likely ancestral area of *T*. *officinale*. This west-to-east scenario of dispersion across Europe and Asia is also supported by tree topologies inferred from sequences of all four loci. Members of Europe and Asian population were sharing common linages in phylogenetic tress in case of *T*. *officinale* (Figs 2 and 3).

The ITS data was used to calculate divergence time among population of Europe and Asian regions of *T*. *officinale*. Divergence time was calculated based on average substitution rate of population of all identified species. ITS substitution rate for asteraceae is 2.51x^10-9^ substitutions per site yer^-1^ based on *Eupatorium* spp.[40] that will obviously give us a rough estimate. This suggests that East Asian population evolved 1.05 million years ago from European parental population (Fig 5).

## Discussion

Dandelions (*T*. *officinale*) are the plants that have been the topic of scientific and medicinal research for more than a century. In this study, plant samples were collected from 16 different countries of the world. We generated sequence datasets of four different barcode loci and used the data for the construction of species relationship, evaluation of divergence and historical biogeography.

An ideal DNA barcode locus must possess short sequence length, to make it easy for PCR, capable to discriminate up to species level and generate sufficient information to separate genomic dataset [41]. The use of combination of barcode loci is considered as the most suitable approach for identification of genera and genetic divergence analysis [42]. The use of two barcode loci was proposed for phylogenetic studies of different plant families in some earlier researches [43]. It was proved to be in-consistent in later studies [44, 45]. In the current investigation, the use of only chloroplast DNA (cpDNA) or genomic DNA was not sufficient to provide better resolution. Previous studies regarding *T*. *officinale* [46, 47] also show weaknesses in species resolution by using only cpDNA. The possible reasons can be low substitution rate and high structural mutations with homoplasy [13]. In view of the results of this study combination of all four loci “ITS2 + matK + rbcL + psbA-trnH” is proposed best for barcoding of *T*. *officinale* (Supplementary table C in S1 Tables). The possible reasons are high percentages of the best match and best close match values, higher PCR success and sequencing rate and ultimately high resolving power of genetic divergence.

The variation of lengths in amplified sequences of different species can be considered possible limitation due to insertion, deletion or genetic re-arrangements [48]. Use of only non-encoding regions are not sufficient in genetic study because of difficulty in logical alignment of obtained genomic sequence loci [49]. Therefore, the sets of barcode loci containing both encoding and non-encoding regions were used in this study. This approach is suitable for both species identification and phylogenetic studies requiring comprehensive sequence alignment. Furthermore, this approach has made it possible to target genetic variations at multiple parts of the genome.

There have been five attempts (including the present study) to construct *Taraxacum* phylogeny with varying barcode loci and sample sizes [8, 13, 46, 47]. Some common findings were the low substitution rates and high length variation with indels presence [13]. Unlike previous studies, plant samples were collected from vast geographical range to uncover maximal variation within the population.

Topological incongruence among genes trees derived from individual gene datasets (SLST approach) was seen at some branches in phylogenetic analysis of *T*. *officinale*. Phylogeny resolution is often effected by discordant gene events [50]. The possible factors can be gene duplication, recombination and incomplete gene sorting [51]. Some recombination events were detected in *T*. *officinale*. This can be the likely reason for this incourgence along with the hybridization process. All haplotypes of *T*. *officinale* were divided into two main groups; Group A and B (Fig 2). The group A comprised of two distinct clades containing haplotypes from China and south Asian regions (Pakistan). Haplotypes from Europe and West Asia region got sister lineage and differentiated in two distinct clades in group B.

In order to infer the connections of *T*. *officinale* population, haplotype analysis was performed based on dataset of all barcode loci. In most of the cases there was considerable levels of population connectivity across different geographical regions (Fig 3). It seems to be a very weak effect of geographical distance on gene flow process. Secondly, the lack of geographical structuring among haplotypes network of *T*. *officinale* represents recent demographic events and a population growth. Large haplo-groups containing members from different continents prove weak population differentiation and large number of recombination events among different population (Fig 3).

Overall, *T*. *officinale* population connectivity seems to be not limited by distance at the European and Asian regions. Detection of a number of re-combination events (Fig 4) among Europe and Asian population further support this hypothesis.

Finally, we implied integrative approach based on all analysis (S-DIVA and MCMC analyses, phylogeny construction, haplotype, recombination and gene flow analysis) in current study and constructed historical biogeography of *T*. *officinale* and related taxa and lineages dispersal patterns. The analysis infers that *T*. *officinale* lineages dispersed from the Europe (putative Taraxacum origin area) toward West Asia and from the west toward the East Asian region (Fig 5). This study suggests West Asia is the geographic center of the distribution range for most of the Taraxacum taxa examined in current study. The presence of less genetic differentiation (F_*ST*_) between European and Asian population of *T*. *officinale* further supports this model. The recombination and gene flow analysis indicate that population currently occurring in east and west Asian countries received a gene flow from Europe. Some potential recombinants of *T*. *officinale* were detected in the East Asian regions with major and minor parents from Europe and Asia (Fig 4). Such dispersal routes have most likely occurred because of suitable climatic conditions that allowed spatial displacement of the *T*. *officinale*.

ITS data was used to calculate divergence time among population of European and Asian regions of *T*. *officinale*. This suggests East Asian population evolved from Europe parental populations nearly 1.05 million years ago from European parental population (Fig 5). As geographical expansion of *T*. *officinale* is dated to the Pliocene. This range expansion may have been triggered by the existence of East Asian winter monsoons during the Pliocene [52–54]

Tajima’s D and Fu’s F_*S*_ are the most powerful tools to detect deviation from neutrality and population expansion scenarios [55]. Both of these analysis provide an insight that multiple selective forces and evolutionary processes have been the targets of selection in *T*. *officinale* evolution. Population with positive values of Tajima’s D represents excess of intermediate frequency alleles and can result from population bottlenecks, structure and balancing selection [56]. Most of the populations had negative values for Fu’s F_*S*_ and Tajima’s D. Either positive selection or recent population expansion is the most likely explanation for these values [27]. Thus we can conclude that multiple selective forces were involved in evolutionary process of *T*. *officinale*. Non-significant values of Tajima’s D and Fu’s F_*S*_ were seen in case of some populations. This can be due to low sample size of given population or simply fits with neutrality. Globally, these results demonstrate that the population differentiation of *T*. *officinale* species was driven by different selective forces and evolutionary processes for different species. This variable range of selection forces may facilitate adaptation to different environments. The findings of Tajima’s D and Fu’s F_*S*_ are also supported by S-DIVA and Bayes MCMC analysis. These analyses inferred both dispersal and vicariance events at different nodes (Fig 5). Regarding vicariance events, the possible explanation can be seed dispersals by waterfowl, likely giving distribution to far isolated areas [57].

The rapid geographic and population expansion of a species is usually facilitated by the changes of landscape and climate factors [58, 59]. Over the time, ecosystem has experienced large variations in environmental conditions linked to trophic interactions and biogeographical shifts of flora and fauna [60–62]. The regimes of climatic change is often reported as in favor of species abundance and biogeographical expansion [63, 64]. Variability and recent changes in environment along with adaptation to the novel local environments may have contributed to the population and biogeographical expansion of *T*. *officinale*. It would have been almost equally possible that favorable environmental conditions, large seed dispersal rate of *T*. *officinale* and fast growth of newly established population helped rapid population expansion next to Europe and West and East Asian regions. As stated earlier the geographical expansion of *T*. *officinale* is dated to the Pliocene and may have been triggered by the existence of monsoons weather.

Another important implication of geographic expansion is the introduction of genetic variation by recombination and gene flow process among different species that has the potential to positively affect population dynamics. Recombination is the source of genetic variation upon which other evolutionary forces can then operate. A recombination event can provide a genetic variant that would require many mutation events to be obtained without recombination [65]. Recombination events can thus impact evolution and population dynamics in different ways. They have been associated with the expansion of host ranges and appearance of new species [66]. It shuffles existing mutations to generate new variants that can then be better adapted to the environment and hence speeding up adaptation process [67]. This study provided the evidence of some potential recombination events among different populations of Taraxacum. Presence of the recombination events would have improved the adaptability of this plant at new geographical localities. Likewise, recombination in Taraxacum can also be associated with the acquisition of novel traits for better adaptation to the new environments. Secondly, recent studies provide a new insight into the adaptation of meiosis during polyploid evolution. Polyploid species can accommodate increased genetic dosage or divergent genomes [68]. The presence of disomic and polysomic inheritance in some *Taraxacum* species reflects positive implications for evolution and genetic improvement to adopt newly inhabited areas.

**Fig 6:**
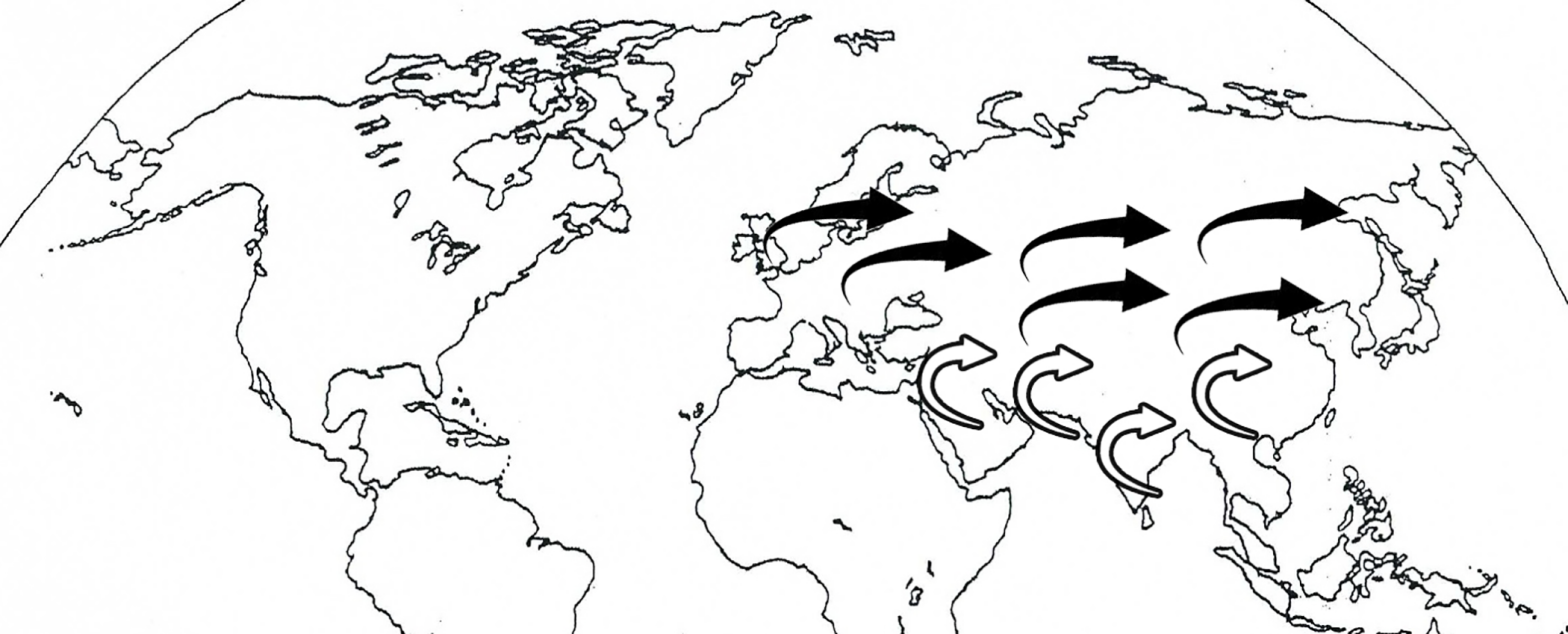
The wind pattern favors *Taraxacum* seed dispersal. Black arrows show westerlies winds whereas white arrows shoe monsoon wind patterns.

Spatial processes are important for determining the dynamics of population and biogeography. Seed dispersal is a leading spatial demographic process among sessile organisms setting the spatial template for population expansion [16, 69]. Recent field studies provide an insight of the seed dispersal patterns and their implications in population evolution and genetics [15]. *Taraxacum* seeds can be dispersed over considerable distances, germinate under a variety of conditions and seedlings survive amongst other plants [70, 71]. Previous studies have shown that a close relative of *Taraxacum* (*Hypochaeris radicata*) successfully dispersed via seed in the Netherlands causing substantial levels of gene flow and population expansion [72]. Seasonal differences in wind directionality and speed greatly effects seed dispersal events [73]. Westerlies are the prevailing winds from the west toward east directions (Fig 6). These winds cover middle latitude where most of the Europe, Middle East and Asian countries are situated. These wind patterns supported the spread of *Taraxacum* population from its putative origin towards East Asian countries. In the same way, the bidirectional wind patterns of Monsoon over Middle East and Asia favor the spatial pattern of seed dispersal between both regions.

## Conclusions

In conclusion, our findings suggest that the genus *T*. *officinale* prevails complex phylogeny and single molecular barcode loci has little power to reveal deep phylogeny. There was considerable incongruence in every gene tree based on individual barcode loci. Use of multi locus sequence typing approach based on datasets of different barcode loci are more efficient in resolving complex *T*. *officinale* phylogeny and evolutionary events. The results of this study indicate evidence for ongoing gene flow and recombination between European and Asian population of *T*. *officinale*. Finally, the intra- and intercontinental expansion of *T*. *officinale* was driven by both biological dispersals and vicariance from Europe to Asian regions. This enigma suggests that environmental factors and evolutionary changes may have been favorable for distribution of *T*. *officinale*.

## Supporting information

S1 Tables (A) = Details of samples collection sites, sample collector’s name and height from the sea. (B) = Details of PCR primers used in this study. (C) = Genetic distance percentage generated using Kimura 2-parameter model analysis for the candidate barcode loci and their combinations. (D) = Details of NCBI Accessions. (E) = A detailed overview of recombination analysis performed by RDP V4 software.
(DOCX)
