# Supplementary material for: Genetic diversity and biogeography of T. officinale inferred from multi locus sequence typing approach
Source: PLoS One. 2018 Sep 18;13(9):e0203275. doi: 10.1371/journal.pone.0203275 (PMC6143195; doi:10.1371/journal.pone.0203275)
Supplement: S1 Tables — (A) = Details of samples collection sites, sample collector’s name and height from the sea. (B) = Details of PCR primers used in this study. (C) = Genetic distance percentage generated using Kimura 2-parameter model analysis for the candidate barcode loci and their combinations. (D) = Details of NCBI Accessions. (E) = A detailed overview of recombination analysis performed by RDP V4 software. (DOCX) [file pone.0203275.s001.docx]

**S1 Table (A).**

| **Name of the place** | **Collector’s Name** | **Height from sea (m)** | **Coordinates** |
| --- | --- | --- | --- |
| Amol-Iran | Adel khashaveh | 63 | 36°28′11″N 52°21′03″E |
| Amsterdam-Netherlands | Matteo cappari | -2 | 52°22′N 4°54′E |
| Basra-Iraq | Yousef jafari | 4 | 30°30′N 47°49′E |
| Beijing-China | Kathy lee | 44 | 39°55′N 116°23′E |
| Belgium-United kingdom | Matteo cappari | 181 | 50°51′N 4°21′E |
| Canton of Bern-Switzerland | Matteo cappari | 1723 | 46°50′N 7°37′E |
| Firozkoh-Iran | Yousef jafari | 1960 | 35°45′25″N 52°46′26″E |
| Gorgan–Iran | Yousef jafari | 1400 | 36°50′19″N 54°26′05″E |
| Hefei-China | Xiahu lee | 37 | 31°52′N 117°17′E |
| Huang pi-China | Kathy lee | 80 | 30°52′30″N 114°22′30″E |
| Huangshi-China | Kathy lee | 27 | 30°13′N 115°05′E |
| Islamabad-Pakistan | Aqueel Ahmad | 620 | 33°43′N 73°04′E |
| Jiuquan-China | Pang yan ju | 1483 | 39°46′N 98°34′E |
| Karbala-Iraq | Yousef jafari | 8 | 32°37′N 44°02′E |
| Karlsruhe-Germany | Matteo cappari | 118 | 49°00′33″N 8°24′14″E |
| Kasur-Pakistan | Aqeel Ahmad | 217 | 31°7′0″N 74°27′0″E |
| Kunming-China | Kathy lee | 1892 | 25°04′N 102°41′E |
| Lahore-Pakistan | Waheed Akram | 217 | 31°32′59″N 74°20′37″E |
| Langroud-Iran | Yousef jafari | 15 | 37°11′49″N 50°09′13″E |
| Lanzhou-China | Pang yan ju | 1320 | 36°02′N 103°48′E |
| Larijan-Iran | Adel khashaveh | 1536 | 35°55′34″N 52°17′12″E |
| Lausanne-Switzerland | Matteo cappari | 495 | 46°31.19′N 6°38.01′E |
| Liaoning-China | Kathy lee | 24 | 41°06′N 122°18′E |
| London-United kingdom | Matteo cappari | 174 | 51°30′26″N 0°7′39″W |
| Lorena-France | Matteo cappari | 184 |  |
| Loshan -Iran | Yousef jafari | 1420 | 36°37′14″N 49°30′38″E |
| Mahmodabad-Iran | Adel khashaveh | -7 | 37°01′59″N 50°19′35″E |
| Nanjing-China | Kathy lee | 22 | 41°06′N 122°18′E |
| Nantes-France | Matteo cappari | 19 | 47°13′05″N 1°33′10″W |
| Noor-Iran | Yousef jafari | 25 | 36°34′25″N 52°00′50″E |
| Karachi-Pakistan | Waheed akram | 8 | 24°51′36″N 67°0′36″E |
| Portugal-Lisbon | Matteo cappari | 2 | 38°46′N 9°9′W |
| Qaemshahr-Iran | Adel khashaveh | 143 | 36°27′47″N 52°51′36″E |
| Qazvin-Iran | Yousef jafari | 1800 | 36°16′N 50°00′E |
| Quito-Ecuador | Saskia | 3000 | 00°14′S 78°31′W |
| Rahimabad-Iran | Adel khashaveh | 1286 | 37°01′59″N 50°19′35″E |
| Rasht-Iran | Yousef jafari | 50 | 37°16′51″N 49°34′59″E |
| Razekeh-Iran | Adel khashaveh | 284 | 36°28′11″N 52°21′03″E |
| Sölden-Austria | Matteo cappari | 1368 | 46°58′N 11°00′E |
| Seoul-South Korea | Xiahou lee | 282 | 37°34′N 126°58′E |
| Sydney-Australia | Kathy lee | 58 | 33°51′54″S 151°12′34″E |
| Taidi Cadore-Italy | Matteo cappari | 878 | 46°27′N 12°23′E |
| Telgte-Germany | Matteo cappari | 54 | 51°58′55″N 7°47′08″E |
| Trentino-Italy | Matteo cappari | 325 | 46°26′44″N 11°10′23″E |
| Urmia-Iran | Adel khashaveh | 1362 | 37°33′19″N 45°04′21″E |
| Urumqi-China | Pang yan ju | 290 | 43°49′30″N 87°36′00″E |
| Wuhan-China | Mohammadjavad Jafari | 20 | 30°35′N 114°17′E |
| Wuwei-China | Pang yan ju | 1790 | 37°55′41″N 102°38′29″E |
| Yicheng-China | Kathy lee | 35 | 31°42′N 112°22′E |
| Yining-China | Hu xiang dong | 666 | 43°55′N 81°19′E |
| [Zhengzhou](https://en.wikipedia.org/wiki/Zhengzhou)-Henan-China | Hu xiang dong | 104 | 34°46′N 113°39′E |

**S1 Table (B).**

| **Primer name** | **Sequence 5-3′** | **Reference** |
| --- | --- | --- |
| rbcL 1F  rbcL 1379R | ATG TCA CCA CAA ACA GAG ACT AAA GC  TCACAAGCAGCAGCTAGTTCAGGACTC | (Kress and Erickson, 2007) |
| matK F  matK R | AYGAAAGYCRYTTAYGGATCT  CGTATTGTACTYCTATGTTTRCCAGC | (Kim et al., 2016) |
| ITS2 GYM_5.8S F2  ITS | GYAGAATCCCGTGARTCATC  TCCTCCGCTTATTGATATGC | (Chen et al., 2010) |
| trnH-psbA TRNH  trnH-psbA psbA | CGC GCA TGG TGG ATT CAC AAT CC  GTT ATG CAT GAA CGT AAT GCT C | (Sang et al., 1997) |

**S1 Table (C).**

| **Barcode loci and combinations** | **Minimum** | **Maximum** | **Mean** |
| --- | --- | --- | --- |
| **ITS2** | 0.00 | 0.94 | 0.31 |
| **matK** | 0.00 | 0.21 | 0.06 |
| **rbcL** | 0.00 | 0.08 | 0.02 |
| **psbA-trnH** | 0.00 | 0.37 | 0.18 |
| **ITS2+ matK** | 0.00 | 0.17 | 0.09 |
| **ITS2+ rbcL** | 0.00 | 0.26 | 0.11 |
| **ITS2+ psbA-trnH** | 0.00 | 0.51 | 0.16 |
| **matK + ITS2** | 0.00 | 0.18 | 0.06 |
| **matK + rbcL** | 0.00 | 0.23 | 0.07 |
| **matK + psbA-trnH** | 0.00 | 0.34 | 0.14 |
| **rbcL+ ITS2** | 0.00 | 0.19 | 0.02 |
| **rbcL+ matK** | 0.00 | 0.51 | 0.21 |
| **rbcL+ psbA-trnH** | 0.00 | 0.78 | 0.39 |
| **psbA-trnH + ITS2** | 0.00 | 0.32 | 0.06 |
| **psbA-trnH + rbcL** | 0.00 | 0.60 | 0.20 |
| **psbA-trnH + matK** | 0.00 | 0.49 | 0.18 |
| **ITS2 + matK + rbcL + psbA-trnH** | 0.00 | 0.83 | 0.36 |

| **ITS** | **rbcL** | **matK** | **psbA-trnH** |
| --- | --- | --- | --- |
| MG519288-322 | MG564359-393 | MG564394-427 | MG582070 -104 |

**S1 Table (D).**

**S1 Table (E)**.

| **Recombinant** | **Major Parent** | **Minor parent** | **GENECOV** | **Max Chi** | **Chimera** | **Si Scan** |
| --- | --- | --- | --- | --- | --- | --- |
| HAP31 | Unknown | HAP15 | **-** | **+** | **-** | **-** |
| HAP2 | HAP10 | Unknown | **+** | **+** | **+** | **-** |
| HAP14 | HAP10 | Unknown | **+** | **+** | **+** | **+** |
| HAP7 | HAP15 | Unknown | **-** | **+** | **+** | **-** |
| HAP19 | HAP25 | Unknown | **+** | **+** | **-** | **-** |
| HAP10 | Unknown | HAP1 | **+** | **-** | **-** | **+** |
